# Starch as a Green Binder for the Formulation of Conducting Glue in Supercapacitors

**DOI:** 10.3390/polym11101648

**Published:** 2019-10-11

**Authors:** Paweł Jeżowski, Przemysław Łukasz Kowalczewski

**Affiliations:** 1 Institute of Chemistry and Technical Electrochemistry, Poznan University of Technology, Berdychowo 4, 60-965 Poznań, Poland; 2 Institute of Food Technology of Plant Origin, Poznań University of Life Sciences, 31 Wojska Polskiego St., 60-624 Poznań, Poland; przemyslaw.kowalczewski@up.poznan.pl

**Keywords:** binder, charge propagation, eco-friendly, energy storage

## Abstract

This work describes the use of commercially available starch as a binder for the preparation of conductive glue and electrode materials. It is demonstrated that starch can be successfully implemented as a binder in energy storage systems with non-aqueous electrolytes. These devices are characterized by a stable cycle life (for 50,000 cycles) at a nominal voltage of 2.5 V. Moreover, the use of starch-based conductive glue improves the electrochemical performance, especially reducing the internal resistance of the device. Starch-bound electrodes display lower equivalent distributed resistance (EDR) values than electrodes using carboxymethylcellulose (CMC) as the binder. This is due to the noticeably lower pore clogging by starch. An electric double-layer capacitor (EDLC) in organic electrolyte (1 mol L^−1^ TEABF_4_ in ACN) at a nominal voltage of 2.5 V can reach a specific power and energy of 100 kW kg^−1^ and 12 Wh kg ^−1^, respectively. This study shows that starch-based conductive glues and electrode materials can be incorporated in EDLC systems.

## 1. Introduction

Electrochemical energy storage devices are composed of two electrodes separated by a porous membrane and soaked in electrolyte. In particular, for electric double-layer capacitors (EDLCs), the energy is stored in an electric double layer (EDL) formed at the electrode/electrolyte interface. Cations and anions in electrolytic solution are attracted to the oppositely charged electrode surfaces, which creates an electric potential difference (voltage). The higher the nominal voltage is (the difference in measured potential between the positive and negative electrode during cell operation), the higher the energy stored (1) and power density (2), according to Equations [1,2,3]:

(1) $$E=C/2\cdot U^{2}$$

(2) $$P=U^{2}/\left(4\cdot ERS\right)$$

where *C* is the capacitance (F), *U* is the nominal voltage (V), and *ERS* stands for the equivalent series, which represents all of the factors contributing to the overall value of resistance (Ω).

To reach high values of energy and power, it is necessary to reach a high nominal voltage and lower the resistance of the cell. Nonetheless, the capacitance part of the equation should not be omitted. The capacitance value increases the overall energy of the constructed device and strongly depends on the electrode materials used and final cell construction, according to the equation provided by Helmholtz [4] (3):

(3) C=(ε·S)/d

where ε is the electric permittivity (F m^−1^), *S* is the accessible surface area [m^2^], *d* is the thickness of the EDL (m), and the accessible surface area *S* is strictly related to the porosity of the electrode material. Equation (3) was developed later by Stern [5], who took into account that the diffusion coefficient at the electrolyte/electrode interphase caused by the agglomeration of differently charged species (ions) near the electrode surface. Calculation of capacitance value for supercapacitors according to the equation (3) has informative value. The theoretical values of electric permittivity found in the literature may differ from the practical values. The accessible surface for ions is not the same as the value of specific surface area (SSA) calculated from gas adsorption isotherms [6]. Moreover, the penetration of pores plays important role especially in the organic electrolytes due to the sieving effect [7]. Additional research and studies were done in this topic to analyze how the capacitance can be theoretically calculated and some mathematical models were proposed in the recent years by Nagy et al. [8] and Huang et al. [9], where the properties of the electrode/electrolyte interphase as well as the material porosity were taken into account.

However, for the practical reasons the formula (4) is used to calculate the capacitance [3]:

(4) C=Q/U=(I·t)/U

where *Q* is the charge accumulated (C), *I* is the current used during charging (A), *t* is the time of the charging process (s), and *U* is voltage (V).

Purely physical energy storage mechanisms are very fast and allow high values of power close to 10 kW kg^−1^ to be achieved [10,11]. To further improve such power values, research has synthesized and used different materials such as activated carbons, carbon cloths, carbon fibers, and other active materials due to their large surface area. However, electrode materials are composed not only of an active material but also a material for increasing the conductivity and a substance binding all components together. Recent reports have described the fabrication of electrode materials with desired properties, such as certain pore sizes and distributions thereof [12,13,14,15,16,17,18], or with the use of specially designed electrolytes [19,20,21,22,23,24,25]. Another approach is to increase the energy by so-called internal hybridization, where a cell is composed of two different electrodes, where one electrode exhibits battery-like behaviour, while the second electrode accumulates charge in an EDL [26,27,28,29,30,31]. To date, however, the impact of the binder has been omitted in the majority of these studies. There are only a few reports concerning the use of novel binders that can be categorized as “green” materials, which have a low impact on the natural environment. Some examples include cellulose [32], carboxymethylcellulose (CMC) [33,34], chitin/chitosan [35,36] and casein [37], just to mention a few. Nevertheless, there is a possibility of preparing so-called binder free electrodes; however, in most cases, for the preparation of electrodes, it is necessary to use sophisticated methods, conditions, or equipment [38,39].

In light of recent studies regarding starch-based binders, which present good cohesive properties, we have decided to pursue the interesting topic of “green” components for electrode preparation. In one study by Passerini [40], it was emphasized that for starch-based electrodes, the “EIS measurements suggest that the coating process needs to be optimized”, which gives space for improvement in this field. One reasonable approach would be to try and modify the contact surface between the current collector and electrode material [41,42,43]. Nevertheless, the etching of current collectors is difficult, and it is necessary to provide strict and harsh conditions to achieve suitable results. Varzi et al. [40] used an etched current collector however, we suggest a completely different approach: an electrode material and conductive glue based on starch, one casted on the other.

Starch is a naturally abundant polymer produced by many plants as a source of energy. It is worth noting that starch is the second most abundant biomaterial in nature. On the one hand, starch is environmentally friendly due to its renewable and biodegradable nature; on the other hand, it is inexpensive and widely available [44,45,46]. Therefore, starch has been used for years not only as a component of adhesives used for gluing paper but also as a binder in many industries [47]. Nevertheless, the binding capacity of starch in its native form is not strong enough [48]; thus, additives that increase its adhesive properties, such as polyacrylamides, polyvinyl alcohol, or polyvinyl acetate, are often used in the corresponding glue formulations [49,50,51,52]. The industrial use of native starch as a binder also limits the difficulty of obtaining an adhesive paste containing a sufficiently high amount of starch (on a dry basis) to create a continuous weld between the bound surfaces [53,54]. In addition, the gelling process, i.e., heating the starch slurry to 60–70 °C significantly increases the viscosity [55,56], making it difficult to pump the adhesive properly in automated production lines and spread it evenly over the surface to be bound. To reduce the viscosity, controlled depolymerization is performed, and appropriately selected auxiliary agents that modify the adhesive properties of the obtained glue are added [57,58].

The abovementioned reasons were the driving force for this work, which seeks to establish a preparation method for a conducting glue based on starch for energy storage devices, which could improve the charge propagation and power performance of electrochemical devices and compete with conductive glues available on the market.

## 2. Materials and Methods 

The conducting glue was prepared by mixing 1:1:1 (mass ratio) deionized water (solvent), starch and soot (Super C65, Imerys, France). The mixture was stirred with a mechanical stirrer at 500 RPM at an elevated temperature of 60 °C. Once gelation of the mixture was observed, the glue was spread on the surface of a current collector. To evenly distribute the conductive glue, stainless steel foil (S316L, thickness 10 μm, Materialis Elements, Wrocław, Poland) was placed on the perforated table of an automatic film applicator with a doctor blade attachment. The current collector sheet covered with conductive glue was transferred to a fume hood, and the excess solvent was evaporated by using a heating plate at 100 °C for 30 min. In the absence of the adhesive glue, extremely low charge propagation and high internal resistance were observed (Figure S1 in Supplementary Materials). The current collector sheet prepared for the CMC-based electrode material was covered with a commercially available conductive glue (Electrodag^TM^ PF-407C, Acheson, Düsseldorf, Germany). The casting and drying method was identical to that described above.

**Figure 1:**
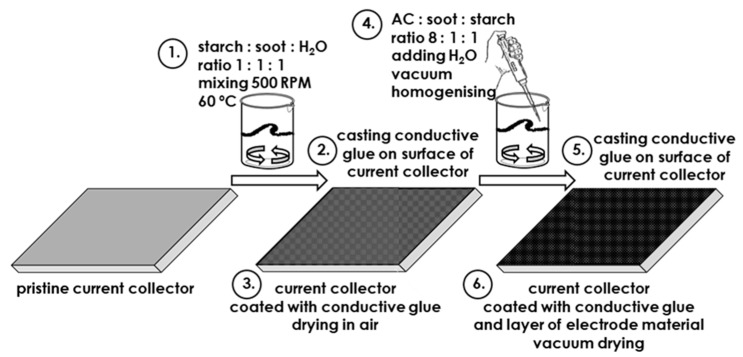
Scheme of the experimental procedure.

The electrode material was prepared according to the following composition: 80 wt. % activated carbon (Kuraray YP 80F, Tokyo, Japan, with characteristic *V*_micro_ < 2 nm = 0.652 and SSA = 2093 m^2^ g^−1^), 10 wt. % carbon black (Super C65, Imerys, France) and 10 wt. % starch or carboxymethyl cellulose (referred to as CMC). Distilled water was added in ratios of 1 mL to 1 g of the dry electrode material mixture to decrease the viscosity until reaching a fluid-like consistency. The mixture was stirred at 20 °C using a vacuum homogenizer until a homogeneous suspension (slurry) was obtained. In the next step, the slurry was spread on the surface of the stainless steel foil covered, with conducting glue being used as the current collector. The gap between the blade and surface of the current collector was adjusted to 100 μm. The sheet of the current collector covered with the conductive glue and electrode material was transferred to a fume hood, and the excess solvent was evaporated using a heating plate at 100 °C for 1 h. Finally, to remove water from the pores of the activated carbon material, the electrode material sheet was dried under vacuum in a dryer (VACUCELL MM44, MMM Medcenter Einrichtungen GmbH, München, Germany) overnight at 100 °C. The dried material was subjected to calendaring to further improve cohesion and adhesion. The final thickness of the electrode material was ca. 70 μm. Electrodes in the form of discs with a diameter of 10 mm were cut out using an EL-CUT precision disc cutter (EL-CELL GmbH, Hamburg, Germany), and the measured mass loading for the electrodes was ca. 2 mg cm^−1^. The scheme of experimental procedure is presented in Figure 1.

The prepared electrodes were analyzed by scanning electron microscopy (SEM) (Quanta 250 FEG, Thermo Fisher Scientific, Waltham, MA, USA) at an accelerating voltage of 5 kV and a probe current of 100 pA.

A portion of the electrode material was used for the estimation of the specific surface area (SSA) and pore size distribution (PSD) according to the Brunauer–Emmett–Teller (BET) equation. SSA was estimated using the accelerated surface area and porosimetry system ASAP 2020 (Micromeritics Instrument Co., Norcross, GA, USA). Degassing of the investigated samples was performed for 24 h at 50 °C under vacuum. Gas adsorption was carried out at −196 °C with N_2_ as the adsorbate.

Swagelok cells were assembled in a glove box (UNILab 1200/780, M. Braun Inertgas-Systeme GmbH, Garching, Germany) with an oxygen and water level below 1 ppm to determine the capacitance value. Electrodes with the same diameter and similar masses were placed in a Swagelok cell. Both electrodes were separated by a porous glass fiber separator (GF/A, Whatman, Darmstadt, Germany, thickness 270 μm), and then electrolyte, one molar solution of tetraethylammonium tetrafluoroborate dissolved in acetonitrile (1 mol L^−1^ TEBF_4_ in ACN, 250 μL), was introduced into the system. A VMP3 potentiostat/galvanostat (Bio-Logic, France) was used for electrochemical testing. The techniques included galvanostatic cycling with potential limitation (GCPL) with a current density up to 50 A g^−1^ (the value was based on the total weight of the electrode material) in the voltage range from 0 to 2.5 V and electrochemical impedance spectroscopy (EIS) in the frequency range from 100 kHz to 10 mHz with an amplitude of 5 mV.

A VMP3 potentiostat/galvanostat (Bio-Logic Science Instruments, Seyssinet-Pariset, France) was used for electrochemical testing. GCPL was performed with a current density of C/20 and C/2 in the potential range from 10 mV to 1.5 V vs. Li^0^/Li^+^ (where C corresponds to the theoretical capacity of graphite, i.e., 372 mAh g^−1^). 

## 3. Results and Discussion

**Figure 2:**
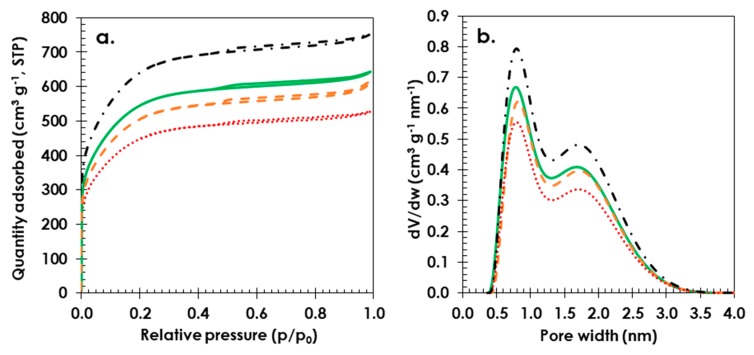
Gas adsorption data: (**a**) adsorption/desorption Brunauer-Emmett-Teller (BET) isotherms and (**b**) pore size distribution (PSD) curves for pristine activated carbon (Kuraray YP 80F) (**dashed-dotted black line**) and electrodes with different binders: starch (**solid green line**), PTFE (**dashed orange line**) and CMC (**dotted red line**). Composition of electrode material 80 wt. % activated carbon (YP 80F) 10 wt. % carbon black (Super C65) and 10 wt. % binder.

The gas adsorption analysis data presented in Figure 2 shows that the highest SSA was observed for the starch-based electrode material (1781 m^2^ g^−1^, solid green line) compared to that of polytetrafluoroethylene (PTFE) (1651 m^2^ g^−1^, dashed orange line), and the lowest value was detected for the CMC (1463 m^2^ g^−1^, dotted red line) bound electrode material. Based on the PSD data (Figure 2b), it seems that starch does not block micropores to the same extent as the PTFE or CMC binders. The starch-based electrode shows a cumulative micropore volume of *V*_micro_ < 2 nm = 0.688, while that for the PTFE-bound electrode material is smaller, with a total micropore volume of *V*_micro_ < 2 nm = 0.505; the value is even smaller for the electrode material with CMC (*V*_micro_ < 2 nm = 0.453). The blockage of pores in AC materials by CMC has been reported by Richner et al., supporting the findings of this study [59]. Pristine AC powder (Kuraray YP80F) (dashed-dotted black line) has been added to Figure 2 as a reference.

**Figure 3:**
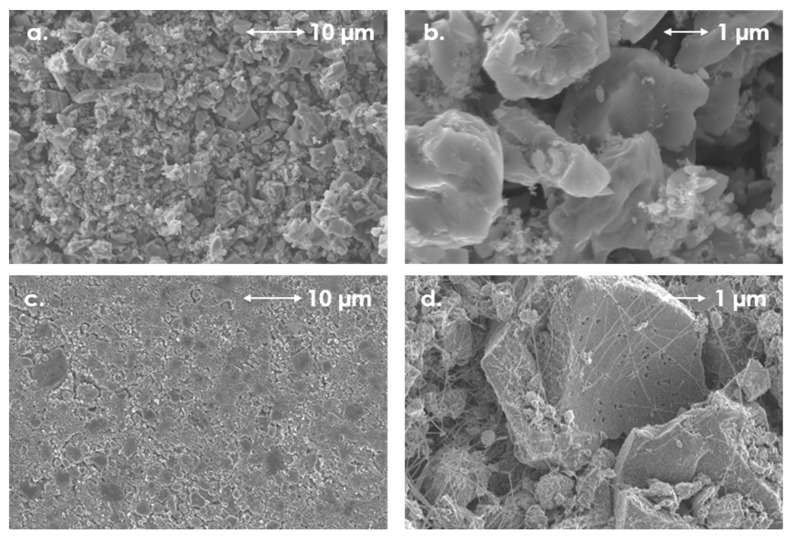

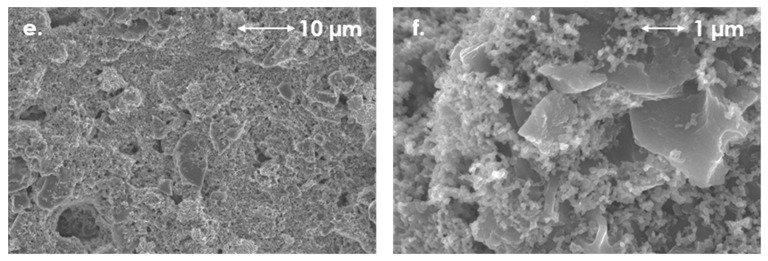
SEM images of electrodes with different binders: starch (**a**,**b**), PTFE (**c**,**d**) and CMC (**e**,**f**), at different magnifications (scale added to the images). Composition of electrode material 80 wt. % activated carbon (YP 80F) 10 wt. % carbon black (Super C65) and 10 wt. % binder.

The SEM images demonstrate the morphologies of the electrode materials with starch (Figure 3a,b), PTFE (Figure 3c,d) and CMC (Figure 3e,f) as binders. In the case of starch and CMC, the morphology of electrode materials is very similar. For PTFE we observed a “spider-web” connections, where threads of PTFE link and bind material particles. In all of the cases, the obtained electrodes were homogenous and the soot particles were well dispersed among the activated carbon particles.

**Figure 4:**
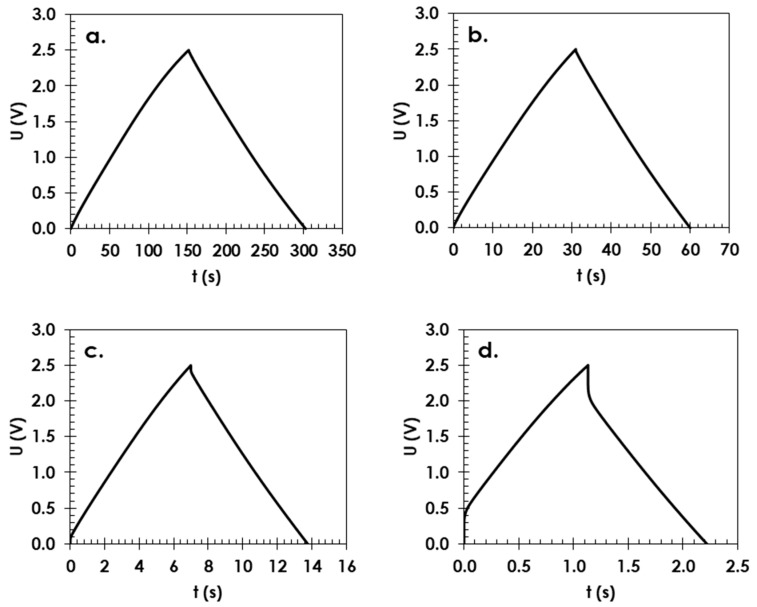
Charge/discharge profiles in the voltage range 0.0 to 2.5 V for EDLCs operating at different current densities: (**a**) 0.5 A g^−1^, (**b**) 2.5 A g^−1^, (**c**) 10 A g^−1^ and (**d**) 50 A g^−1^, with 1 mol L^−1^ TEABF_4_ in ACN as the electrolyte. Composition of electrode material 80 wt. % activated carbon (YP 80F) 10 wt. % carbon black (Super C65) and 10 wt. % starch.

Figure 4 presents charging/discharging voltage profiles for EDLCs with starch-bound electrode materials coated on the surface of starch-based conductive glue. The triangular shape of these profiles and coulombic efficiency near 100% indicate no parasitic reactions in the presented system. For the lower current densities (0.5 A g^−1^), the lower efficiency at the initial cycles can be caused by the functional groups on surface of activated carbon. However, after several cycles it reaches the columbic efficiency of nearly 100% (Figure S2 in Supplementary Materials). For the higher current densities for example 50 A g^−1^ the observable ohmic drop (IR drop) is related with the internal resistance of the cell which increases when the value of passing current increases [60,61,62]. Moreover, in Figure S3 (in Supplementary Materials), we present additional data obtained for starch-bound electrodes operating in aqueous electrolyte (1 mol L^−1^ Na_2_SO_4_). However, this subject still needs additional work regarding the use of different types of starch and their modifications, which could prevent the dissolution of starch in water-based electrolytes for EDLCs during electrochemical cycling.

**Figure 5:**
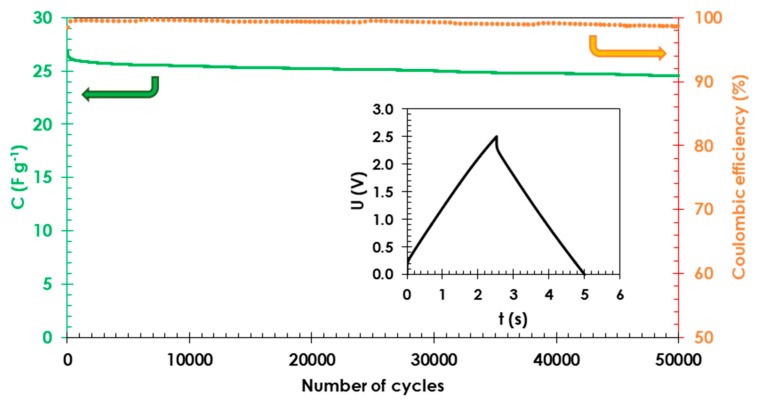
Cyclability of EDLC cells with starch binder in the voltage range from 0.0 to 2.5 V at a current density of 25 A g^−1^ and 1 mol L^−1^ TEABF_4_ in ACN used as the electrolyte. Capacitance values and columbic efficiency are represented as a solid green line and dotted orange line, respectively. Composition of electrode material 80 wt. % activated carbon (YP 80F) 10 wt. % carbon black (Super C65) and 10 wt. % starch.

To establish the long-term stability of the electrode materials with starch binders, cells were continuously charged and discharged at a current of 25 A g^−1^ in a voltage range from 0.0 to 2.5 V. The table capacitance value (green solid line) during 50,000 cycles (capacitance retention of 95% compared to the initial value before cycling) and high coulombic efficiency of 98%, as shown in Figure 5, suggest that starch can be used as a binder for electrode materials and as a conductive glue in EDLCs with long lifespans.

**Figure 6:**
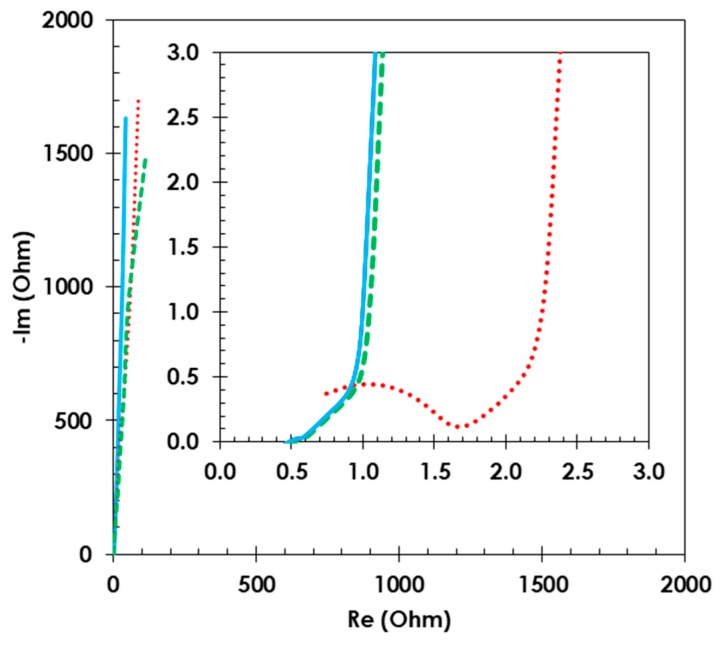
Comparative Nyquist plots for EDLCs with starch as an electrode material binder before (**solid blue line**) and after cycling life studies (**dashed green line**) and with CMC as a binder (**dotted red line**). Composition of electrode material 80 wt. % activated carbon (YP 80F) 10 wt. % carbon black (Super C65) and 10 wt. % binder. 1 mol L^−1^ TEABF_4_ in ACN was used as the electrolyte.

Nyquist plots (Figure 6) show that the EDLC constructed with conductive glue and electrodes incorporating the starch binder has an equivalent distributed resistance (EDR) = 0.9, which is lower than for CMC-bound electrodes, where the EDR value is 2.2 Ω.

**Figure 7:**
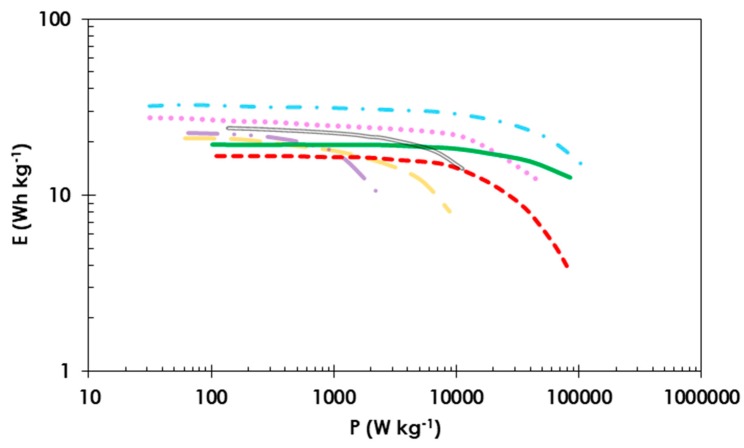
Comparative Ragone plot for EDLC cells in which starch (**solid green line**) or CMC (**dashed red line**) was used as a binder. Cells were tested up to 2.5 V in 1 mol L^−1^ TEABF_4_ in ACN. Composition of electrode material 80 wt. % activated carbon (YP 80F) 10 wt. % carbon black (Super C65) and 10 wt. % binder. The reference curves were made according to the data from: [63] (**dot-dashed light blue line**), [64] (**double grey line**), [65] (**dotted light pink line**), [66] (**long dashed light orange line and dot-dot-dashed light purple line**).

The two EDLC systems operating at 2.5 V with starch or CMC as the binder are compared in a Ragone plot (Figure 7). The starch-based EDLC has a higher specific energy of 20 Wh kg^−1^ than the EDLC with CMC-bound electrodes (17 Wh kg^−1^). At high power values (>10 kW kg^−1^), the energy density of the cell with commercially available conductive glue and CMC binder starts to decrease, most likely because of the higher internal resistance; thus, the specific energy is 4 Wh kg^−1^ at a specific power of 100 kW kg^−1^. In the case of the EDLC utilizing starch-bound electrodes and conductive glue, it is possible to reach a high specific energy of 12 Wh kg^−1^ at a specific power of 100 kW kg^−1^. Obtained results were compared with other data found in the literature about symmetric electrochemical capacitors with carbon electrodes. All of the cells operate in organic electrolyte with TEABF_4_ where salt concentration is 1 mol L^−1^ [63,64,65,66]. It shows that the use starch conductive glue allows us to reach high values of power without a noticeable fade of energy, especially when reaching the value of specific power 10 kW kg^−1^.

## 4. Conclusions

Starch can be considered as a “green” binder material for the fabrication of conducting glue and electrodes for energy storage devices. The manufacturing process does not involve any toxic or harmful solvents. The electrochemical characteristics of electrode materials incorporating starch as a binder and a layer of starch-based conductive glue provide better results than the commonly used “green” binder, CMC. The use of relatively high current loads, such as 50 A g^−1^, is possible for cells where the electrode material is fixed to the current collector with a starch-based conducting glue, and such cells exhibit better charge propagation, excellent capacitance retention and relatively small resistance. Moreover, preliminary studies have shown that starch binder can be used in aqueous electrolytes, which will be investigated further in the future.

## Supplementary Materials

The following are available online at https://www.mdpi.com/2073-4360/11/10/1648/s1, Figure S1: Comparative Nyquist plots for EDLC with starch as a binder in conductive glue for improved adhesion of electrode material to current collector (green solid line) and without adhesive conductive layer (red dotted line). Composition of electrodes 80 wt. % activated carbon (YP 80F) 10 wt. % carbon black (Super C65) and 10 wt. % starch. Figure S2: Cyclability of EDLC cells with starch binder in the voltage range from 0.0 V to 2.5 V at a current density of 0.5 A g^−1^ and 1 mol L^−1^ TEABF4 in ACN used as the electrolyte. Capacitance values and columbic efficiency are represented as a solid green line and dotted orange line, respectively. Composition of electrodes 80 wt. % activated carbon (YP 80F) 10 wt. % carbon black (Super C65) and 10 wt. % starch. Figure S3: Electrode potential profiles and cell voltage of EDLC operating with aqueous electrolyte (1 mol L^−1^ Na_2_SO_4_) at current density 1.0 A g^−1^. Composition of electrodes 80 wt. % activated carbon (YP 80F) 10 wt. % carbon black (Super C65) and 10 wt. % starch.
