# Supplementary material for: Starch as a Green Binder for the Formulation of Conducting Glue in Supercapacitors
Source: Polymers (Basel). 2019 Oct 11;11(10):1648. doi: 10.3390/polym11101648 (PMC6836256; doi:10.3390/polym11101648)
Supplement: Supplementary file 1 [file polymers-11-01648-s001.pdf]

Starch as a Green Binder for the Formulation of Conducting Glue in Supercapacitors

Paweł Jeżowski * and Przemysław Łukasz Kowalczewski

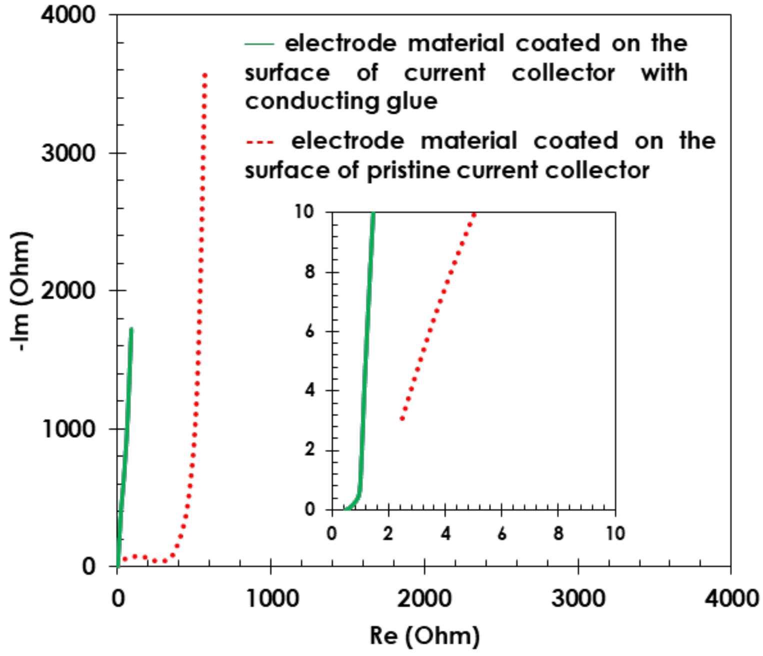

Figure S1. Comparative Nyquist plots for EDLC with starch as a binder in conductive glue for improved adhesion of electrode material to current collector (green solid line) and without an adhesive conductive layer (red dotted line). Composition of the electrode material: 80 wt. % activated carbon (YP 80F), 10 wt.% carbon black (Super C65), and 10 wt.% starch. The electrolyte was 1 mol L^{-1} $TEABF_4$ in ACN.

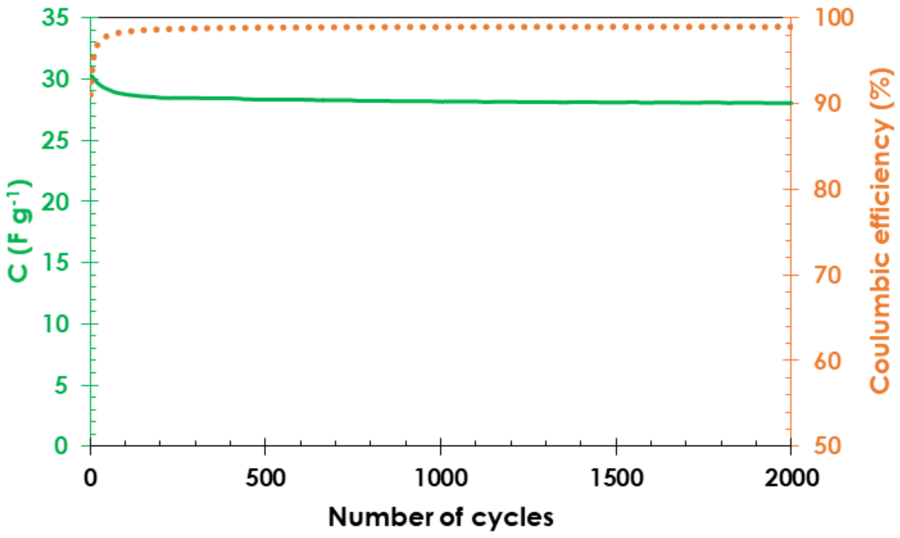

Figure S2. Cyclability of EDLC cells with starch binder in the voltage range from 0.0 to 2.5 V at a current density of 0.5 A g^{-1} and 1 mol L^{-1} $TEABF_4$ in ACN used as the electrolyte. Capacitance values

and columbic efficiency are represented as a solid green line and dotted orange line, respectively. Composition of the electrode material: 80 wt % activated carbon (YP 80F), 10 wt % carbon black (Super C65), and 10 wt % starch.

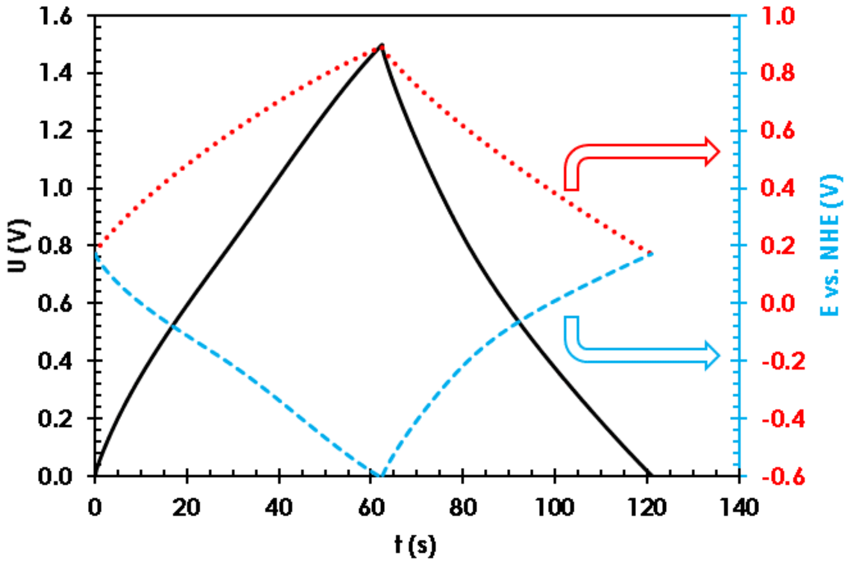

Figure S3. Electrode potential profiles and cell voltage of EDLC operating with aqueous electrolyte (1 mol L^{-1} Na_2SO_4) at current density 1.0 A g^{-1}. Composition of the electrode material: 80 wt. % activated carbon (YP 80F), 10 wt. % carbon black (Super C65), and 10 wt. % starch.
